# The Microenvironmental Effect in the Progression, Metastasis, and Dormancy of Breast Cancer: A Model System within Bone Marrow

**DOI:** 10.1155/2012/721659

**Published:** 2012-02-06

**Authors:** Bobby Y. Reddy, Philip K. Lim, Kimberly Silverio, Shyam A. Patel, Brian Wong Won, Pranela Rameshwar

**Affiliations:** Department of Medicine-Hematology/Oncology, New Jersey Medical School, University of Medicine and Dentistry of New Jersey, 185 South Orange Avenue, Newark, NJ 07103, USA

## Abstract

Despite diagnostic advances, breast cancer remains the most prevalent cancer among women in the United States. The armamentarium of treatment options for metastatic disease is limited and mostly ineffective with regards to eradicating cancer. However, there have been novel findings in the recent literature that substantiate the function of the microenvironment in breast cancer progression and the support of metastasis to tertiary sites such as bone marrow. The uncovered significance of the microenvironment in the pathophysiology of breast cancer metastasis has served to challenge previously widespread theories and introduce new perspectives for the future research to eradicate breast cancer. This paper delineates the current understanding of the molecular mechanisms involved in the interactions between breast cancer cells and the microenvironment in progression, metastasis, and dormancy. The information, in addition to other mechanisms described in bone marrow, is discussed in the paper.

## 1. Introduction

The ability to invade and metastasize allows cancer cells to leave sites of primary tumor formation and recolonize in new tissues. This offers immediate metastasis to distant sites as well as the establishment of dormancy. Metastases are responsible for approximately 90% of human cancer deaths [1]. The previously established theory on metastasis described the phenomenon as a process alike to the Darwinian evolution [2]. In that perspective, cancer cells undergo a process of natural selection which favors rare cells within a tumor capable of invading and growing at sites of metastasis. The natural selection was believed to involve the development of stable genetic alterations which proffer the potential for successful metastasis. However, advances in technology, especially the development of high-throughput microarray expression profiling and *in vivo* imaging, have served to challenge this perspective of cancer metastasis [2]. Research suggests that metastatic ability is gained at earlier stages of tumor expansion than predicted by the previous model, and that this ability is acquired through transient changes in gene expression. A new tumor microenvironment invasion model reconciles the Darwinian perspective with recent discoveries. The tumor microenvironment consists of surrounding stroma, which is composed of extracellular matrix and various cell types including endothelial cells, fibroblasts, and infiltrative leukocytes. 

The microenvironment, in addition to providing a scaffold for the organ, has been found to play a significant role in breast cell function through paracrine, mechanical, and hormonal interactions [3]. In the tumor microenvironment invasion model, stable genetic changes in primary tumor cells induce the microenvironment to initiate transient changes in gene expression which promote invasiveness and metastasis. Hence, the tumor microenvironment invasion model predicts that selected mutations within primary cancer cells drive the microenvironment to induce transient and epigenetic changes required of metastasis [2, 4]. This model is supported by *in vivo* imaging of mammary tumors, which demonstrates the following regarding motile tumor cells: they represent only a small percentage of tumor cells, they are distributed throughout the tumor, and they are found most commonly localized to precise areas within the tumor [5]. Furthermore, genes associated with metastasis are expressed early and are found in tumor cells throughout the tumor [2]. Also in support of the model is the observation that micrometastases are commonly genetically heterogeneous, indicating that the invasiveness and migration are not limited to stable gene alterations.

Dormant cancer cells can remain quiescent for >10 years. Cancer can resurge and metastasize to tertiary organs. However, similar dormancy can occur in other organs. This paper will discuss on the bone marrow biology and describe how cancer cells could take advantage of the bone marrow microenvironment to adapt a dormant phenotype. Dormancy is defined as a state of fully transformed cells with nontumorigenic property that resists anticancer agents. Clinical dormancy has been defined as the time (5–25 yrs) between removing the primary tumor and relapse [6]. We expand this definition by proposing that dormant breast cancer cells exist in bone marrow and other organs long before clinical detection of the tumor [7].

We focus on bone marrow mostly due to its implication as the source of tumor-initiating cells in a large number of breast cancer resurgence [8, 9]. Also, prognosis is worse when breast cancer cells micrometastasize to the bone marrow [10]. An understanding of the mechanisms by which the bone marrow microenvironment facilitates a dormant phenotype of breast cancer cells is significant for strategies to target dormant breast cancer cells with minimum toxicity.

Bone marrow stromal cells, which are located close to the endosteum, support breast cancer cell quiescence as well as resurgence [11–15]. Quiescence is partly explained by the production of cytokines from stroma and gap junctional intercellular communication between the cancer cells and stroma [13, 16, 17]. Gap junction facilitates the passage of microRNA (miRNA) between the cancer cells and stroma [16]. Among these miRNAs are those that target CXCL12, which pass from stroma to breast cancer cells [16, 17].

 Although the idea of crosstalk between the tumor and the microenvironment to promote growth and metastasis is now generally accepted in the field of cancer biology, the mechanisms underlying the interactions has not been well established. For example, in the primary site, the quantities and components of the microenvironment vary among tumors [18]. Though tumors require stroma for maintenance and growth, the malignant potential of a tumor does not correlate with the amount of surrounding stroma; both highly and less malignant cancer cells can have abundant or scarce surrounding stroma [18]. Rather, the microenvironmental effects on tumor progression are attributable to complex and dynamic epigenetic and phenotypic alterations. In addition to contributing to cancer progression and metastasis, the microenvironment may also play a pivotal role in protecting cancer cells from immune surveillance and response. In this paper, we delineate the current understanding of the microenvironemental involvement in breast cancer progression, metastasis, and dormancy in the mammary gland and then extrapolate the results to dormancy in bone marrow.

## 2. Composition of the Microenvironment

Stromal-epithelial interactions have been implicated in breast cancer progression [19, 20]. The composition of the tumor stroma is different from bone marrow stroma. The whole bone marrow-supporting stroma are mostly fibroblasts, in other organs, the tumor stroma consists of a heterogeneous population of cells, including pericytes, tumor-associated macrophages, epithelial cells, endothelial cells, fibroblasts, myeloid-derived suppressor cells, and adipocytes [21, 22]. Although each component might serve a unique role in facilitating the growth of breast cancer at the primary site, the stromal components are likely to interact to support and protect the tumor. Interestingly, these same cellular elements can be located at sites of distant metastasis, where they serve to provide a supportive niche. Reciprocal interactions between breast cancer cells and tumor stroma at the primary site govern the behavior of cancer [23]. This is explained by the report showing the secretion of soluble factors from the cancer cells to activate the surrounding stromal cells. Consequently, the stromal cells respond to promote invasiveness of the breast cancer cells [24].

Tumor-associated macrophages constitute a major immune cell population within the tumor microenvironment and play an important role in chronic inflammation during cancer progression [25]. Within the tumor-associated macrophage population, there is a high level of plasticity in terms of function [25, 26]. Primarily, the macrophages stimulate the formation of new blood vessels in the tumor bed via the production of vascular endothelial growth factor [27]. In addition, the macrophage can also induce a state of local immunosuppression, which can provide the tumor with an advantage to survive within the immune system [25]. The role of macrophages is complex since these cells can also promote the invasiveness of cancer via matrix remodeling through the secretion of matrix metalloproteases MMP7 and MMP9 [28]. Remodeling of the tumor stroma can also occur through the production of CCL18 from tumor-associated macrophages, which accelerates the invasive properties of breast cancer [29].

 The role of adipocytes in the primary tumor microenvironment has been studied recently in an effort to determine the effects of obesity on cancer progression. Coculture of adipocytes with breast cancer cells resulted in adipocyte activation and secretion of MMP11, as well as proinflammatory cytokines IL-6 and IL-1*β* [24]. The increased production of IL-6 from cancer-associated adipocytes promotes breast cancer cell invasion [24]. Since obesity results in poor prognosis of breast cancer [30] and adipose tissues are a source of mesenchymal stem cells [31], studies on adipose cells are relevant to the well-established interaction between mesenchymal stem cells and breast cancer cells [32]. Mesenchymal stem cells, through the production of interleukin-6, can enhance breast tumor growth [23].

Additional role of mesenchymal stem cells are included in this section. The role of fibroblasts within the breast tumor microenvironment as cellular support for cancer cells is not mutually exclusive of mesenchymal stem cells. Soluble factors from tumors are thought to differentiate mesenchymal stem cells into myofibroblast, which produce stromal cell-derived factor-1 (SDF-1) to accelerate breast cancer growth [33]. The mechanisms underlying this interaction have been determined to be hepatoma-derived growth factor and cyclophilin B from the tumor-conditioned media [34]. In addition, carcinoma-associated fibroblasts can alter the local T-cell balance by polarizing towards a Th2-type response, and this resulted in the loss of the antitumor Th1 effects [35]. This immune switch is not only limited to the differentiated mesenchymal stem cells. Studies with bone marrow mesenchymal stem cells showed similar findings, in addition to increases in regulatory T cells and reduced production of granzyme B to induce cytotoxicity [36]. 

The myeloid-derived suppressor cells can also protect the tumors from the immune system [37]. Myeloid suppressor cells are a heterogeneous collection of immune cells with immune-inhibitory properties [38]. Their numbers are increased in the circulation of patients with breast cancer as compared to healthy controls [39]. Although the studies on myeloid-derived suppressor cells in breast cancer are relatively limited, this area is a rapidly expanding area of cancer research. Recent findings demonstrate that the myeloid suppressor cells are capable of interfering with the activation of antitumor T-cell responses. Interestingly, interluekin-12, with antitumor activity [40], has been shown to decrease the number of myeloid-derived suppressor cells in the tumor microenvironment [38], underscoring another mechanism by which cells within the tumor microenvironment can protect the cancer cells from the immune response.

Overall, this section provides an overview of the tumor microenvironment at the primary site, with a diverse group of cells that promote and protect tumors. The majority of cells, however, appear to play key roles in breast cancer growth at the primary sites. The bidirectional crosstalk between breast cancer cells and microenvironmental components cannot be overlooked, since cellular interactions *in vivo* have a strong influence on the biological behavior of cancer cells. The significance of these findings points to an important role for stromal-epithelial interactions in overall breast cancer progression and metastasis. A recent review paper describes that a shift in the microenvironment can lead to the tumor and how this information can be explored for clinical intervention [20]. 

## 3. Mechanical Interactions

Although the interactions between tumor cells and stroma through cytokines and other soluble factors has received significant attention in the literature, the less familiar topic of mechanical interactions is also important to cancer progression and metastasis. Cells within tissue are under constant physical forces from neighboring cells and surrounding extracellular matrix (ECM), and these forces can be in the form of shear stress, compression, or tension. These forces from the microenvironment can serve to initiate mechanical signaling pathways after being perceived by mechanically responsive sensors present throughout the cell [18]. This signaling can subsequently induce changes at the molecular levels which promote cell survival, division, and motility. For example, an important family of mechanotransducers is the integrins, plasma membrane proteins which interact externally with ECM and internally with components of the cytoskeleton [18]. Integrins can undergo force-dependent activation resulting in the formation of focal adhesions, which can serve to induce growth and migration [41]. During the development of breast cancer, tension homeostasis is significantly perturbed [18]. There are amplified compression forces secondary to pressure from the progressively enlarging mass, matrix tightening from desmoplastic changes, and elevated interstitial pressure from leaky vasculature and compromised lymphatic drainage [18]. This state of abnormal force leads to the disruption of cell-cell junctions and polarity, and these changes collectively promote anchorage-independent survival and invasion. Also, the compression stress can lead to tumor angiogenesis directly through increasing VEGF-A expression or indirectly by generating hypoxic conditions through disrupting existing vasculature around the tumor, which also ultimately leads to increased VEGF-A expression [18]. Furthermore, exceeding compression force significantly reduces surrounding interstitial space, which allows for abnormal accumulation of fluid from leaky vasculature and blocked lymphatic drainage. This fluid tends to contain concentrations of cytokines and growth factors much greater than physiologic levels, promoting aggressive tumor expansion and migration. In addition, the overwhelming interstitial pressure can also serve to obstruct access of chemotherapeutic medications to the tumor. In summary, the mechanical influences of the microenvironment are extremely important to carcinogenesis and metastasis, and hence this topic warrants further investigation. 

## 4. Epithelial-to-Mesenchymal Transition (EMT)

**Figure 1:**
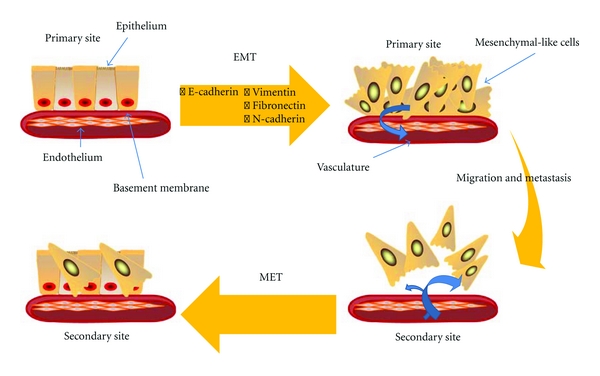
The epithelial-to-mesenchymal transition (EMT) is a physiological process by which an epithelial cell loses polarity and assumes a mesenchymal phenotype. While EMT can occur naturally in gastrulation and wound repair, it is involved as a route of metastasis in cancer. Through molecular changes, such as the loss of E-cadherin, the epithelial cell undergoes remodeling and loosens its attachments from the basement membrane and adjoining cells to enter the vasculature. Once mobile, the malignant cells can take up residence at secondary sites, reverting to an epithelial cell type or remaining dormant.

EMT is a complex phenomenon that is believed to play a role in dormancy and metastasis. EMT is a normal physiologic process during embryogenesis, wound healing and repair, and tissue remodeling [42]. EMT is characterized by the loss of epithelial polarity and the subsequent development of a fibroblast-like phenotype (Figure 1) [43]. The precise mechanisms of EMT in breast cancer remains uncertain, but it is believed to involve diverse changes at the genetic and molecular levels. Phenotypically, EMT involves the loss of epithelial cell markers such as E-cadherin, *γ*-catenin, zonula occludens-1 (Zo-1), and the acquisition of mesenchymal markers, such as vimentin, fibronectin, and N-cadherin [43]. The role of N-cadherin in promoting invasion, and migration of cancer cell has been established [44]. Moreover, the upregulation of EMT markers is correlated with poor prognosis [44]. An examination of the cell qualities of epithelial and mesenchymal cells demonstrates how EMT promotes cancer metastasis. Epithelial cells are organized tightly together to form a continuous layer above a basement membrane, while mesenchymal cells are loosely anchored and have the capability of becoming motile [45]. 

 The microenvironment can trigger EMT through induction via upregulation of specific cytokines and growth factors. TGF-*β* is known to be a potent inducer of EMT, particularly during the early stages of carcinogenesis [43]. Also, phorbol myristate acetate (PMA) can initiate EMT through the activation of protein kinase C [46]. Furthermore, the microenvironment can influence EMT through facilitating inflammation and accompanying leukocyte migration. Inflammation-associated EMT involves epigenetic changes induced by the increased expression of NF-*κβ*, Src, microRNAs, and IL-6 [3]. The mechanism through which CD8+ T cells can induce EMT involves the induction of CD44+/CD24− stem cell-like phenotype in breast cancer cells, which promotes invasiveness and metastasis, along with resistance to chemotherapy [3].

 EMT is a particularly important area of microenvironment-breast cancer crosstalk because it is a process that can be potentially inhibited by therapeutic intervention. Several agents have shown promise with regards to inhibition of cancer progression associated with EMT. For example, Withaferin-A, a biologically active inhibitor of vimentin, has been found to suppress the mesenchymal phenotype through the induction of apoptosis, while preventing angiogenesis [47]. Also, Klf4, a well-known activator of E-cadherin, has also been found to inhibit EMT and associated invasive potential of transformed BCCs [43]. Inhibitors of the phosphatidylinositol 3-kinase (PI3K)/Akt/mTOR signaling, such as phosphatidylinositol ether lipid analogs and rapamycin, have been also found effective in suppressing EMT [48]. Hence, these preliminary findings demonstrate the promising therapeutic potential of EMT modulators. 

## 5. Dormancy

**Figure 2:**
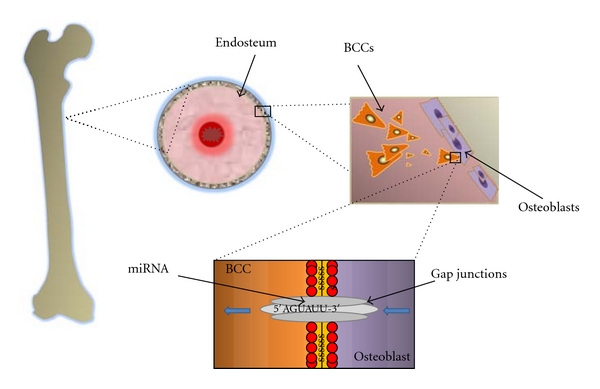
Stromal cells in the endosteal region of the bone marrow produce the chemokine, CXCL12, a known regulator in hematopoiesis. Through an interaction between CXCL12 and CXCR4 (a receptor on the BCC), malignant cells are drawn from circulation to the stromal niche. There, BCCs may form gap junctions with osteoblasts, which facilitates the intercellular transfer of small molecules such as miRNAs. Experimental evidence demonstrates that micro-RNAs can traverse gap junctions and induce dormancy of BCCs.

A significant challenge of breast cancer treatment is the transition of cancer cells to a dormant phenotype. The literature supports that breast cancer relapses from bone marrow years after remission, suggesting a preferential niche in the bone marrow microenvironment for circulating tumor cells [49]. Dormant cells are arrested at the G1 phase of cell cycling. Quiescence proffers cancer cells with survival advantage through resistance to chemotherapeutic agents, which are designed to target proliferating cells [49]. Experimental evidence suggests that dormant cancer cells exist in the bone marrow near the endosteum, where they form gap junctional intercellular communication (GJIC) with hematopoietic-supporting cells and stroma (Figure 2) [50]. Connexin 43 (Cx43) is involved in the formation of GJIC between breast cancer cells and stroma [16]. An important factor of the breast cancer cell-stroma crosstalk in the bone marrow is CXCL12, a chemokine that interacts with CXCR4 and CXCR7 [31]. CXCL12 is normally constitutively generated by stroma, but it is downregulated when breast cancer cells contact stroma [17]. A decline in CXCL12 production correlates with decreased breast cancer cell proliferation [17]. A recent study identified certain microRNAs (miRNAs) which cross GJICs between breast cancer cells and stroma and specifically reduce CXCL12 levels [16]. In this study, 4 miRNAs were found to traverse GJICs and transition BCCs to the G_0_ phase of the cell cycle [16]. These novel findings suggest that microRNAs may play an integral role in breast cancer dormancy in the bone marrow. Furthermore, these data offer significant promise for developing treatment options targeting dormant cancer cells. Currently, there is an ongoing phase I clinical trial using siRNA to treat patients with solid cancers; hence, targeting miRNAs may also be a plausible treatment strategy in the near future [51].

 The interaction between mesenchymal stem cells (MSCs) and BCCs in the bone marrow microenvironment is also implicated in dormancy. It has been found that BCCs interact with MSCs through CXCL12-CXCR4 upon traversing blood vessels in the bone marrow [49]. The mechanism through which MSCs offer protection to BCCs is hypothesized to involve the immunosuppressive properties of MSCs [42]. MSCs have been found to induce the production of regulatory T cells (T_regs_) when cocultured with BCCs, which allows BCCs to evade immune response [52]. This concept of MSCs preventing the eradication of cancer cells from physiologic antitumor immune responses is termed oncoprotection [42]. The involvement of MSCs in breast cancer and other cancers is rapidly expanding area of basic science research, which is bound to lead to promising discoveries. The development of therapies aimed at eliminating MSC-related oncoprotection will be challenging, given the ubiquitous existence of MSCs and their relevance to many important biological functions. However, if further research uncovers specific distinctions in MSCs involved in oncoprotection, compared to normal MSCs, then the potential for therapy will certainly be more promising.

## 6. Conclusion

Studies on the microenvironment of breast cancer are rapidly growing. Novel findings in the recent literature demonstrate the significance of the microenvironment in the progression, metastasis, and dormancy of breast cancer. The objective for scientists, going forward, is transforming the data gained from basic science research into effective therapeutic options. However, the precise mechanisms through which the microenvironment induces molecular alterations in cancer cells remain yet to be elucidated. Also, the parallels of pathologic microenvironmental interactions and physiologic roles pose significant challenges to developing treatment strategies free of adverse side effects. Therefore, further investigations aimed at deciphering the intricacies of the microenvironment need to be performed to optimize therapeutic development.

## Abbreviations

BCC: Breast cancer cell
ECM: Extracellular matrix
VEGF: Human leukocyte antigen
EMT: Epithelial-to-mesenchymal transition
GJIC: Gap junctional intercellular communication.
